# EARLY OUTCOMES OF ROBOTIC ENHANCED VIEW TOTALLY EXTRAPERITONEAL VENTRAL HERNIA REPAIR: A SINGLE-CENTER EXPERIENCE

**DOI:** 10.1590/0102-6720202400032e1825

**Published:** 2024-09-13

**Authors:** Rodrigo PILTCHER-DA-SILVA, Pedro San Martin SOARES, Beatriz Carolina Schuta BODANESE, Gabriel JASINSKI, Ana Carolina de Oliveira MAKIYAMA, João Rafael Bora RUGGERI, Júlio Cezar Uili COELHO, Christiano Marlo Paggi CLAUS

**Affiliations:** 1 Hospital Nossa Senhora das Graças, General and Digestive Surgery Department - Curitiba (PR), Brazil;; 2 Universidade Federal do Rio Grande do Sul, Postgraduate Program in Medicine and Surgical Sciences - Porto Alegre (RS), Brazil;; 3 Universidade Federal de Pelotas, Postgraduate Epidemiology Department - Pelotas (RS), Brazil;; 4 Hospital Nossa Senhora das Graças, Registered Nurse of Robotic Surgery - Curitiba (PR), Brazil.

**Keywords:** Hernia, Hernia Ventral, Incisional Hernia, Robotics, Hérnia, Hérnia Ventral, Hérnia Incisional, Robótica

## Abstract

**BACKGROUND:**

Incisional hernia (IH) is an abdominal wall defect due to a previous laparotomy, and surgical repair is the only treatment. IH has a negative impact on patients’ quality of life. In the last decades, the approach has improved from open to laparoscopic and robotic surgery with the objective of promoting better abdominal wall function after reconstruction. Today, robotic enhanced-view totally extraperitoneal (reTEP) is one of the most advanced techniques for abdominal wall reconstruction.

**AIMS:**

The aim of this study was to analyze the early results of patients with incisional hernia submitted to repair with reTEP.

**METHODS:**

This is a retrospective cohort study, and all patients who underwent reTEP surgery for ventral hernia in the years 2021 and 2022 were included. The only exclusion criteria were patients who underwent another type of herniorrhaphy. Statistical analysis was performed using the Stata software.

**RESULTS:**

A total of 32 participants were submitted to reTEP; the majority had an incisional hernia, and according to the European Hernia Society, EUS-M score 3 was the most prevalent. The mean surgical time was 170 min, and the console time was 142 min. Most patients stayed 2 days in the hospital. No intraoperative complications were reported.

**CONCLUSIONS:**

reTEP is a safe and effective technique and has favorable outcomes in the early postoperative period. Further studies with larger sample sizes and longer follow-up periods are needed to confirm these findings.

## RESUMO

**RACIONAL:**

A hérnia incisional (HI) é um defeito da parede abdominal decorrente de laparotomia prévia e o reparo cirúrgico é o único tratamento. A HI tem impacto negativo na qualidade de vida dos pacientes. Nas últimas décadas, a abordagem evoluiu da cirurgia aberta para a laparoscópica e robótica com o objetivo de promover melhor função da parede abdominal após a reconstrução. Hoje, a visão robótica aprimorada totalmente extraperitoneal (reTEP) é uma das técnicas mais avançadas para reconstrução da parede abdominal.

**OBJETIVOS:**

Analisar os resultados precoces de pacientes portadores de hérnia incisional submetidos à correção com visão robótica aprimorada totalmente extraperitoneal (reTEP).

**MÉTODOS:**

Estudo de coorte, retrospectivo, em que foram incluídos todos os pacientes submetidos à cirurgia reTEP para hérnia incisional, em 2021 e 2022. Os únicos critérios de exclusão foram pacientes submetidos a outro tipo de herniorrafia. A análise estatística foi realizada utilizando o software Stata.

**RESULTADOS:**

Dentre os 32 pacientes submetidos ao reTEP, a maioria apresentava hérnia incisional, e o escore EHS-M 3 foi o mais prevalente. O tempo cirúrgico médio foi de 170 minutos e o tempo de console foi de 142 minutos. A maioria dos pacientes permaneceu 2 dias no hospital. Não foram relatadas complicações intraoperatórias.

**CONCLUSÕES:**

A técnica reTEP é segura e eficaz e teve resultados favoráveis no pós-operatório imediato. Mais estudos com amostras maiores e períodos de acompanhamento mais longos são necessários para confirmar esses achados.

## INTRODUCTION

An incisional hernia (IH) is an abdominal wall defect that occurs at the site of previous surgical scars, commonly in the anterior abdominal wall, and is classified as a type of ventral hernia (VH)^11^. IH develops after laparotomy at an approximate rate of 20%, which could be three times higher in patients with a high risk. This is an important cause of absence from work and has a significant impact on the quality of life (QoL). The only treatment for IH is surgical repair^11^.

In the 1990s, laparoscopic ventral hernia repair (LVHR) with intraperitoneal onlay mesh (IPOM) placement was developed, which promoted a shorter length of stay, reduction in surgical site infections, and similar recurrence rates when compared to the open approach^1^ ^,^ ^4^ ^,^ ^7^ ^,^ ^12^ ^,^ ^15^. However, there were concerns about this technique, particularly related to traumatic mesh fixation, poor mesh integration, and the potential adhesions of the mesh with small bowel or other abdominal viscera even with the use of coated or composite meshes^3^ ^,^ ^4^. As a result, abdominal core health surgeons explored the anatomy and spaces of the abdominal wall for better repair and mesh placement.

Only in 2011 was the enhanced-view totally extraperitoneal (eTEP) technique introduced into the surgeon’s armamentarium, making laparoscopic access to the retrorectus space possible. This technique was first used for inguinal hernia repair and later expanded to VHR^5^ ^,^ ^6^ ^,^ ^10^. Minimally invasive surgery (MIS) for abdominal wall reconstruction became possible by matching the concepts of Rives Stoppa, transversus abdominis release (TAR), and eTEP, enabling hernia repair with appropriate overlap and without visceral contact and penetrating fixation^14^ ^,^ ^15^.

However, due to ergonomic limitations and difficulties in suturing and closing the fascial defect at laparoscopy, Beliansky et al. recently described robotic eTEP (reTEP), a technique for robotic VHR (RVHR). The advantages of robotic systems in VHR include an increase in degrees of freedom, ergonomic improvement, and better performance of delicate movements^5^ ^,^ ^6^. This has brought reTEP into discussion by experts around the world. This recent evolution in hernia surgery shows that surgeons need to be lifelong students if they want to provide the best outcomes for their patients.

The aim of this study was to evaluate the early outcomes of patients who underwent reTEP in our institution. We reviewed all cases operated on in the past 2 years, assessing the perioperative period and early recovery.

## METHODS

A retrospective cohort study was conducted at Hospital Nossa Senhora das Graças in Curitiba (PR), Brazil. The hospital medical records were searched for patients who underwent reTEP surgery for ventral hernia in the years 2021 and 2022. The authors assessed the medical records, and the variables analyzed are presented in Table 1. All patients who underwent ReTEP for VHR were considered eligible. A minimal 3-month follow-up was required. Patients presenting non-midline hernias were excluded from this study. Descriptive statistics were performed using the Stata software. All patients signed the consent form to be included in this study.

**Table 1 -:** Variables.

Patient characteristics	Sex (Male/Female); Age (Years); BMI (kg/m^2^); Comorbidity (No/Yes); Alcoholism (No/Yes); Smoking (No/Yes); DM (No/Yes); SAH (No/Yes); AMI (No/Yes); CI (No/Yes); Arrhythmia (No/Yes); Cirrhosis (No/Yes); CRF (No/Yes); COPD (No/Yes); Stroke (No/Yes); Continuous medication (No/Yes); Antiaggregant (No/Yes); Anticoagulant (No/Yes); Chemotherapeutic (No/Yes); Corticosteroid (No/Yes); Immunobiological (No/Yes); Immunosuppressive (No/Yes); ASA classification (1, 2, and 3).
Hernia characteristics	Hernia type (Umbilical, Epigastric, and Incisional, Umbilical plus bilateral inguinal hernia); Hernia size: Transverse length (mm) and Longitudinal length (mm); Number of defects (One, Two, Three, Four, or more); EHS M (M1 - subxiphoid; up to 3 cm below, M2 - epigastric, M3 - umbilical; 3 cm above or below); EHS W (W1 up to 4 cm, W2 4 up to 10 cm, W3 >10 cm); Width straight right rectus abdominis muscle; Width straight left rectus abdominis muscle; Diastasis (No/Yes); Diathesis size (mm).
Surgical considerations	Surgical time (min); Docking time (min); Access side (Right side of abdomen and Left side of abdomen); Crossover (No/Yes); Transversus abdominis release (No/Yes); Anterior fascia sheath closure V-Loc™ 0; Stratafix 2-0; Stratafix 0; V-Loc™ 1; Stratafix 1); Posterior fascia sheath closure (No/Yes); Mesh (yes); Mesh length (mm); Mesh width (mm); Mesh fixation (No/Yes); Drain (No/Yes); Drain removal time (days); Associated procedures (No/Yes); Spine surgery (No/Yes); Bilateral inguinal hernia (No/Yes); Intraoperative complication (No/Yes).
Postoperative considerations	Complications in the early postoperative period (No/Yes); Bleeding with conservative management without the need for transfusion Ileus (No/Yes); Length of hospital stay (days); First PO appointment (days); Second appointment (30 PO days); Hospital readmission (No/Yes) Seroma (No/Yes); Hematoma (No/Yes); Wound infection (No/Yes); Surgical site infection (No/Yes); Hernia recurrence (No/Yes); Clinical complication - Aritmia; AMI; Pneumonia; DVT; Embolia pulmonary; UTI; BS; Paresthesia; Atelectasis; Dead (No/Yes).

BMI: body mass index; SAH: systemic arterial hypertension; DM: diabetes mellitus; AMI: acute myocardial infarction; CI: cardiac insufficiency; CRF: chronic renal failure; COPD: chronic obstructive pulmonary disease; ASA: American Society Anesthesiology; EHS M: European hernia society classification-midline hernia; EHS W: European hernia society classification - width; V-Loc™: wound closure device; DVT: deep vein thrombosis; UTI: urinary tract infection; BS: brain stroke; PO: postoperative period.

### Surgical considerations

First, by means of a computed tomography (CT) study, the largest retrorectal space is chosen to start the procedure. Thus, our surgical approach begins with an incision close to the costal margin and access to the retrorectus space with an optical laparoscopic trocar. The laparoscopic camera is then used to create the retrorectus space by blunt dissection. Three robotic trocars are placed just medial to the linea semilunaris with care to preserve the neurovascular bundles.

The robot (DaVinci Xi) is then attached, and we continue with the dissection. In most of our cases, the crossover maneuver is initiated cranial to the hernia defect, except in the European Hernia Society (EHS) classification, EHS M1 (subxiphoidal) or high M2 (epigastric) hernias. This step needs to be decided case-by-case since the better place for crossovering is it is far from the hernia to avoid opening the peritoneum and/or having a visceral injury.

Once the crossover is done, the hernia is then dissected and reduced. If the peritoneum opens, it is sutured with polydioxanone 3-0. To avoid opening the peritoneum, it is important, mainly at the beginning of the dissection, once pneumoperitoneum decreases the operative field. The need for transverse abdominal release is evaluated and done if necessary, based on previous CT and tension over the closure of both the posterior peritoneum and anterior fascia. Although it is still not a surgical step defined as essential, we recently began to suture the posterior fascial sheath using a polydioxanone 2-0 barbed suture in cases in which there is not much tension on the closure. The anterior fascial defect is sutured using a polyester 0 or 1 barbed suture, including plication of the diastasis of the rectus muscles when it is present. Thus, the medium-weight polypropylene mesh is placed in the retrorectus spaces without any fixation. A closed-suction drain is placed, and the procedure is ended.

## RESULTS

The present study was composed of 32 participants who underwent reTEP. Table 2 shows the characteristics of the study sample. The casuistic consisted mostly of men (76.1%). The median age was 60.4 years (standard deviation (SD): 13.6). The average body mass index was 28.6 (SD: 13.0). More than half of the participants (64.0%) had at least one comorbidity, with diabetes mellitus and systemic arterial hypertension being the most prevalent, affecting 25.6 and 41.6% of the sample, respectively. None of the participants reported alcohol or tobacco use. Additionally, none of the participants had a history of heart attack, congestive heart failure, arrhythmia, cirrhosis, chronic kidney disease, chronic obstructive pulmonary disease (COPD), or stroke.

**Table 2 -:** Patient characteristics (n=32).

Variable	n (%)
Sex (Male)	24 (76.8)
Age (years; mean, SD)	60.4 (13.6)
BMI (mean, SD)	28.6 (13.0)
Comorbidity (Yes)	20 (64.0)
Alcoholism	0 (0)
Smoking	0 (0)
Diabetes mellitus (Yes)	8 (25.6)
Arterial hypertension (Yes)	13 (41.6)
Heart attack (Yes)	4 (12.8)
Heart failure (Yes)	0 (0)
Arrhythmia (Yes)	0 (0)
Cirrhosis (Yes)	0 (0)
Renal chronic disease (Yes)	0 (0)
COPD (Yes)	0 (0)
Stroke (Yes)	0 (0)
Continuous medication (Yes)	15 (48)
Antiaggregant (Yes)	7 (22.4)
Anticoagulant (Yes)	1 (3.2)
Chemotherapeutic (Yes)	1 (3.2)
Corticosteroid (Yes)	0 (0)
Immunobiological (Yes)	1 (3.2)
Immunosuppressive (Yes)	0 (0)
ASA classification	
1	10 (32.0)
2	18 (57.6)
3	4 (10.4)

BMI: body mass index; COPD: chronic obstructive pulmonary disease; SD: standard deviation; ASA: American Society of Anesthesiology.

About the characteristics of the hernia (Table 3), all patients had an incisional hernia, and 51.2% of the patients had more than one abdominal wall defect. Out of the total number of patients, five (18.5%) had a recurrence of either a previous umbilical hernia repair (3) or a previous incisional hernia repair (2). None of them had the actual surgery performed by the same surgeon as the previous repair. Additionally, all of these patients submitted to open onlay herniorrhaphy in the previous repair.

**Table 3 -:** Hernia characteristics (n=32).

Variable	n (%)
Hernia type	
Incisional	32 (100)
Number of defects	
One	16 (51.2)
Two	7 (22.4)
Three	2 (6.4)
Four or more	2 (6.4)
EHS M	
M1 - subxiphoid (up to 3 cm below)	2 (6.4)
M2 - epigastric	6 (19.2)
M3 - umbilical (3 cm above or below)	24 (76.8)
EHS W	
W1 up to 4 cm	15 (48.0)
W2 4 up to 10 cm	14 (44.8)
W3 >10 cm	3 (9.6)
Width straight right rectus abdominis muscle (mm; mean, SD)	76 (10.8)
Width straight left rectus abdominis muscle (mm; mean, SD)	76.5 (16.5)
Diathesis (Yes)	23 (73.6)
Diathesis size	52.9 (11.9)

EHS M: European hernia society classification - midline hernia; EHS W: European hernia society classification - width.

The EHS-M (midline hernia) score 3 was the most prevalent, reaching 76.8%, and the EHS-W (hernia width) score 1 was found in 48.0% of cases. A total of 66.7% of the patients had rectal diastasis associated with hernia. The TC measurement of the width of the straight right rectus abdominis muscle in a straight line had a mean of 76.00 mm (SD: 10.78) and 76.50 mm (SD: 16.48) on average on the left side. All patients had at least 50 mm of rectus sheath width bilaterally to perform the procedure. Finally, the diastasis size had a mean of 52.9 mm (SD: 11.9) and was corrected in all cases.

The sample consisted of 32 patients with symptomatic hernia without criteria for urgent surgery, all of whom underwent surgery with crossover technique and mesh placement. The mean surgical time was 170 min (SD: 31.9), and the mean docking time was 142 min (SD: 29.0). The majority of patients had American Society of Anesthesiology (ASA) - ASA 1 or 2 classification, with only 4 patients classified as ASA 3. The most common access was by the left side (71.2%). The posterior sheath was closed in 36.0% of patients. The mesh was not fixed in 87.2% of patients, and a drain was used in 73.3% of patients. The mean hospital stay was 2.2 days (SD: 2.1). Two patients (6.4%) experienced complications in the early postoperative period. One patient developed a hematoma that required percutaneous drainage without major consequences, and another patient presented with ileus. On the first outpatient follow-up, one case of seroma with small volume and without repercussions was identified, which was absorbed spontaneously. At a minimum follow-up of 3 months, no other complications were reported, and there were no recurrences.

## DISCUSSION

Since the complexity of abdominal wall surgery has increased, techniques must constantly seek better surgical and aesthetic results that promote less impact on patient QoL. The main concern in hernia follow-up has been hernia recurrence for decades, which has decreased with mesh and a better understanding of the abdominal wall over the years, regardless of technique - open VHR (OVHR), LVHR, or RVHR^17^. In recent years, the concept of abdominal wall function and QoL has grown in importance, becoming as important as objective clinical criteria, such as recurrence rates and surgical site infection or occurrence^11^. In the present study, there was no recurrence, despite a short follow-up.

This evolution with the aim of treating complex hernias and offering better functionality of the abdominal wall has begun with OVHR, followed by LVHR, eTEP, and finally, reTEP, to achieve results in a large number of patients. The eTEP technique is a safe and effective approach for VHR with good aesthetic and abdominal wall function results^1^. Nonetheless, an expert surgeon is needed due to the challenge of creating a retromuscular space and the need for advanced laparoscopic skills for maneuvers in the limited space^1^. Therefore, the most important benefit of reTEP is the robot’s versatility, which extends the possibility of MIS to more complex cases^15^ ^,^ ^16^ ^,^ ^17^.

When comparing MIS to OVHR, it is well-documented that there is an improvement in wound-related morbidities, a reduction in the length of hospitalization, less postoperative pain, faster recovery, and a quicker return to usual activities^9^ ^,^ ^14^ ^,^ ^15^ ^,^ ^17^. In our series of cases, the length of hospital stay was around 2 days, and there was only one case of hematoma requiring percutaneous drainage and another patient with postoperative ileus in the early postoperative period, suggesting early benefit from MIS. There were no complications related to the mesh, and only a small seroma without repercussions was identified on the first postoperative appointment. In the 1-year follow-up evaluation by Zayan et al.^17^, patients who underwent RVHS and robotic inguinal hernia surgery had better scores on the Carolinas Comfort Scale, a disease-specific QoL questionnaire, compared to those who underwent laparoscopic surgery for the same pathologies.

Nonetheless, not all studies comparing LVHR and RVHR have demonstrated any significant differences in perioperative outcomes^8^. Data suggest that patients who underwent RVHR use less pain medication and return to normal activities sooner than those who underwent either OVHR or LVHR^11^. The operative time is still longer in RVHR in most research; however, it is associated with the surgeon’s learning curve for most hernia researchers. Our mean console time was 142 min, which is in accordance with the literature as shown by Morrell et al.^13^, which was 143.8 min (range 75-277). The results may be influenced by the expertise and previous high volume in laparoscopic hernia surgery. It is also important to emphasize that in most cases the hernias were less than 6-8 cm wide (selection bias in the learning curve not of the technique itself but of the robotic platform), although concomitant correction of recti diastasis was performed in most cases.

We understood that laparoscopic eTEP requires experienced hands to be performed, and the robotic platform improved surgical performance. With our first cases, we experienced this change. The benefits of the robotic platform include a three-dimensional view, an increase in degrees of freedom, an improvement in surgeon ergonomics, tremor filtering, and better performance of delicate movements^11^ ^,^ ^16^. In VHR, all of these results in increased dexterity for intracorporeal suturing of the linea alba, which is one of the most important surgery steps and can be difficult even in the hands of experienced laparoscopists^6^. Robotic surgery for hernia repair is an evolution of laparoscopic surgery, enabling MIS for the treatment of a larger number of patients and making possible fascial closure and mesh fixation without the use of transabdominal sutures and tackers^2^ ^,^ ^11^. With our cases, although a small number, we realized that MIS was offered to a larger number of patients, and some of these cases would have been approached by OVHR to obtain adequate overlap if we did not have the robotic platform. The laparoscopic technique was already widely used in our service, which certainly facilitated the retromuscular approach in rETEP, enabling reduced console time and improved outcomes.

## CONCLUSIONS

The study shows that reTEP is a safe and effective technique for the treatment of symptomatic hernias. These findings suggest that reTEP is a viable option for the treatment of symptomatic hernias especially associated with recti diastasis, with favorable early outcomes. Further studies with larger sample sizes and longer follow-up periods are needed to confirm these findings. As with any other new procedure, case selection during the initial experience seems essential for good results. Finally, the use of a robotics platform for complex abdominal reconstruction facilitated the repair and improved the surgical performance.
